# Insights into the molecular determinants involved in *Mycobacterium tuberculosis* persistence and their therapeutic implications

**DOI:** 10.1080/21505594.2021.1990660

**Published:** 2021-11-01

**Authors:** Hemant Joshi, Divya Kandari, Rakesh Bhatnagar

**Affiliations:** a Molecular Biology and Genetic Engineering Laboratory, School of Biotechnology, Jawaharlal Nehru University, New Delhi, India; b Amity University of Rajasthan, Jaipur, Rajasthan, India

**Keywords:** *Mycobacterium tuberculosis*, persistence, *in vitro* stress models, tuberculosis biomarkers, therapeutic approaches, antibiotic tolerance, resuscitation, host immune system

## Abstract

The establishment of persistent infections and the reactivation of persistent bacteria to active bacilli are the two hurdles in effective tuberculosis treatment. *Mycobacterium tuberculosis*, an etiologic tuberculosis agent, adapts to numerous antibiotics and resists the host immune system causing a disease of public health concern. Extensive research has been employed to combat this disease due to its sheer ability to persist in the host system, undetected, waiting for the opportunity to declare itself. Persisters are a bacterial subpopulation that possesses transient tolerance to high doses of antibiotics. There are certain inherent mechanisms that facilitate the persister cell formation in *Mycobacterium tuberculosis*, some of those had been characterized in the past namely, stringent response, transcriptional regulators, energy production pathways, lipid metabolism, cell wall remodeling enzymes, phosphate metabolism, and proteasome protein degradation. This article reviews the recent advancements made in various *in vitro* persistence models that assist to unravel the mechanisms involved in the persister cell formation and to hunt for the possible preventive or treatment measures. To tackle the persister population the immunodominant proteins that express specifically at the latent phase of infection can be used for diagnosis to distinguish between the active and latent tuberculosis, as well as to select potential drug or vaccine candidates. In addition, we discuss the genes engaged in the persistence to get more insights into resuscitation and persister cell formation. The in-depth understanding of persistent cells of mycobacteria can certainly unravel novel ways to target the pathogen and tackle its persistence.

## Graphical abstract

**Figure:**
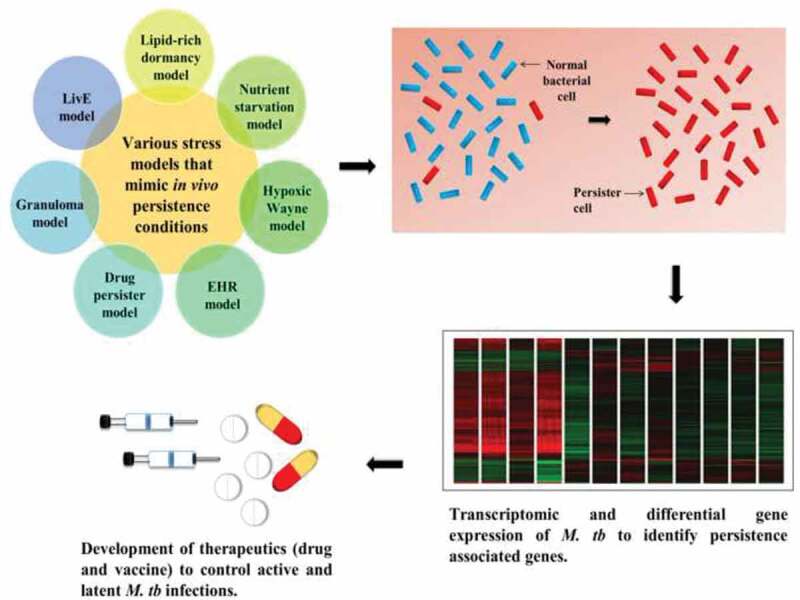

## Introduction

Tuberculosis (TB) remains a serious human health problem caused by *Mycobacterium tuberculosis* (*M. tb*). According to the annual Global TB reports, nearly 10 million people got inflicted with the disease in 2019, and 1.2–1.3 million infected people died [1]. The current therapeutic regimen of tuberculosis includes a combination of four drugs, namely, isoniazid, rifampicin, ethambutol, and pyrazinamide, for a minimum of six months, which is extendable up to nine months in cases of latent bacilli [2]. Although the recommended TB treatment kills most of the drug-susceptible tuberculosis bacteria, a subpopulation remains able to tolerate this long course of treatment. Two key reasons for the failure of therapeutic programme are: the long duration of the treatment and the presence of a large number of asymptomatic TB carriers (Latent TB patients). The dormant or persistent bacteria are believed to switch to an active form whenever the conditions are favorable due to the weakened immune status of the host and are primarily liable for the recalcitrance of mycobacterial infection [3,4]. Persisters are basically the phenotypic variants to wild type bacterial populations that are slow-growing, transiently tolerant to high doses of antibiotics, and may reinitiate the TB infection after cessation of the antibiotic treatment [5,6]. Besides, under antibiotic stress conditions also, the number of persistent bacteria increases, which may be the prominent reason for the failure of the clinical treatment. Despite several efforts and recent advancements in the arena of drugs and therapeutics, there is no drug available that can target the persistent bacteria due to a lack of comprehensive understanding of the molecular mechanism of persistence and reactivation [7]. However, existing studies indicate that persisters arise either stochastically or in response to *in vitro* environmental stress, which mimics the *in vivo* granuloma environment the bacteria encounters during infection [8].

Interestingly, the mechanisms of persister formation via stochastic gene expression or environmental stresses are different. One good example of stochastically induced persistence in *M. smegmatis* to isoniazid where a stochastic decrease in the expression of KatG, an enzyme required for the conversion of isoniazid prodrug to its active form results in the reduction of the effective concentration of the active drug for action, leading to increased tolerance to the drug [9,10]. On the other hand, environmental stress induces a bet-hedging persistence in the preexisting persisters in a bacterial population, which means under stressful conditions, heterogeneity arises in terms of the underlying persister phenotype. Increased tolerance to ciprofloxacin in *E. coli*, a DNA gyrase inhibitor, happens when DNA damage induces TisB expression and the resulting decrease of proton motive force leads to a state of dormancy [10,11].

Further, understanding the molecular mechanisms that control the formation of persisters and their resuscitation has remained elusive. In this review, we describe a comprehensive overview of the *in vitro* stress models that mimic the *in vivo* conditions to identify the molecular determinants of *M. tb* crucial for survival in adverse conditions. Moreover, in-depth information about the genes involved in *M. tb* persistence will pave the way in identifying novel drug or vaccine targets and will help in the eradication of tuberculosis infection by targeting both latent and active *M. tb* and shortening the duration of tuberculosis treatment. Finally, we will briefly discuss the recent developments in drugs and vaccines that target latent mycobacteria to control TB infection.

## Various stress models for studying *M. tb* persistence

**Figure 1.**
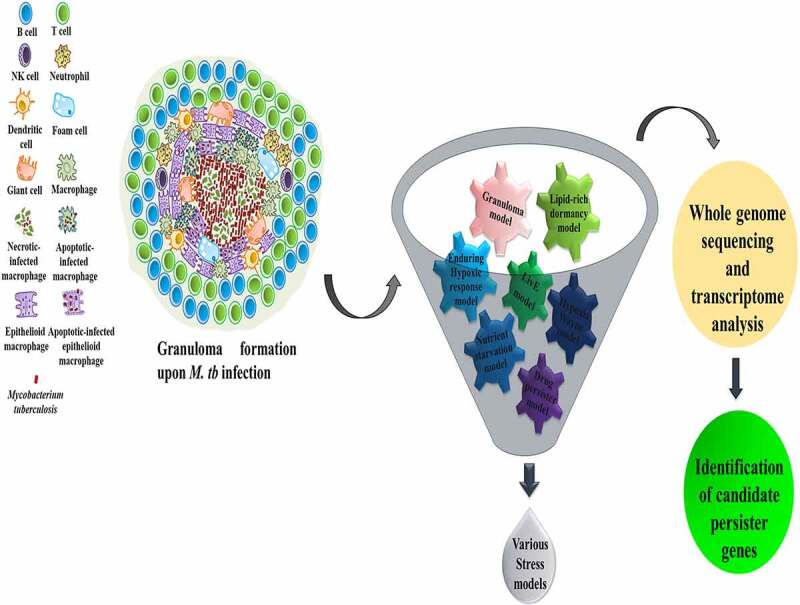
The different stress models imitating the granuloma formed in the host infected with *M. tb*, aid to identify the putative genes involved in the persistence of pathogen

Upon infection into the host, *M. tb* encounters stressful conditions like low oxygen availability, acid stress, nutrient deprivation, and oxidative stress. To gain an insight into the expression of *M. tb* genes against these host-derived stress conditions, *in vitro* stress models that could imitate the *in vivo* stress conditions of infection (as shown in Table 1) along with transcriptomics and whole-genome sequencing approaches are being used by researchers worldwide [7]. The putative candidate genes derived from these approaches are then investigated for their possible involvement in the persistence of *M. tb*. In previous studies using these *in vitro* stress models (Figure 1), putative genes correlated with persistence were predicted but, only a subset of those genes were experimentally proven to be involved [12,13]. Therefore, these models require significant upgradation for them to be employed to understand persistence.

**Table 1.** Various stress models generated to study the *M. tb* persistence with their detailed information

S. no.	Stress model	Type of model	Stress conditions	Description	References
1.	Nutrient starvation model	*In vitro*	Nutrient deprivation	Gene expression profiling of an exponential-phase culture of *M. tb* nutrient starved in phosphate buffer saline for 96 hours.	[14–16]
2.	Hypoxic Wayne model	*In vitro*	Gradual oxygen depletion, stationary phase	Gene expression analysis of *M. tb* in stationary phase and non-replicating persistence phase.	[18,19]
3.	Enduring Hypoxic Response (EHR) model	*In vitro*	Consistent oxygen depletion	Transcriptomic profiling of *M. tb* exposed to consistent oxygen depletion for four and seven days.	[20]
4.	Drug persister model	*In vitro*	Antibiotics stress	Transcriptome analysis of two weeks old culture of *M. tb* treated with D-cycloserine (DCS) antibiotic for 14 days.	[23]
5.	Granuloma model	*Ex vivo*	Hypoxia, oxidative stress, nutrient starvation, acidic pH, antibiotic stress	Gene expression analysis of *M. tb* infected to peripheral blood mononuclear cells (PBMCs) present in extracellular matrix^1^ in presence of rifampicin antibiotic.	[25]
6.	Lysosomal *in vitro* exposure (LivE) model	*Ex vivo*	Hypoxia, nitric oxide, iron limitation, acidic pH, nutrient starvation, stationary phase	Transcriptomic sequence analysis of *M. tb* exposed to the lysosomal soluble fraction extracted from activated bone marrow-derived macrophages (BMMOs).	[26,27]
7.	Lipid-rich dormancy model	*In vitro*	Lipid-rich (cholesterol and fatty acids) environment, hypoxia, stationary phase	Differential gene expressions of *M. tb* in presence of lipid and dextrose, over three different growth phases including exponential phase, stationary phase, and non-replicating persistence phase of hypoxia.	[29]

### Nutrient starvation model

This model mimics a condition of the persistent state of *M. tb* and thus assists in investigating the significance of nutrient availability in granuloma and its effect on *M. tb* persistence. Initially, Loebel et al. (1933) devised this model wherein bacterial cultures were transferred from nutrient-rich to phosphate-buffered saline, which resulted in the gradual depreciation in respiration rates, but the bacteria remained viable and could recover into their active states in the nutrient-rich medium [14,15]. Later this model was modified such that there was cessation of *M. tb* replication and the transcriptomic profile at 96 h revealed that 279 genes were upregulated and 323 genes were downregulated [16]. Furthermore, the downregulated genes were found to be related to energy metabolism, lipid biosynthesis, amino acid synthesis, translation and posttranslation modifications, DNA replication, and virulence, which is quite in tune with the long-term persistence observed in *M. tb*. Later, Jamet et al. (2015) observed that under nutrient starvation conditions stringent response got activated to regulate the genes of mycolic acid biosynthesis (i.e. *hadABC*) that may facilitate the adaptation of *M. tb* to persist for longer periods [17].

### Hypoxic Wayne model

The Wayne model could mimic the “hypoxic TB granuloma environment” through a gradual decrease in oxygen conditions, hence also known as the gradual hypoxic model. In this model, the bacteria are at a non-replicating and low metabolic activity state to persists for longer duration [18]. The transition from aerobic to anaerobic culture conditions are brought by the transfer of growing *M. tb* culture to sealed tubes, for creating a hypoxic environment, as through bacterial metabolism occurs a gradual decrease in oxygen levels. Further under these culture conditions a non-replicative persistence (NRP) state, namely, NRP1 (1% dissolved oxygen saturation) and NRP2 (0.06% dissolved oxygen saturation) [18] reaches. Additionally, the comparison of gene expression profiles of stationary phase and non-replicating persistence phase cultures demonstrates numerous genes induced in the NRP compared to 29 genes of the stationary phase. According to recent studies, five genes that are highly expressed in the NRP model, such as *Rv0251c, Rv0841c, Rv1874, Rv2332*, and *Rv2660c*, along with *Rv3290c* (*lat*) gene that was found to be strongly induced in both stationary and NRP phases [19]. In-depth studies on these results may assist to uncover the bacterial mechanisms for long-term persistence.

### Enduring hypoxic response model

As the name suggests this model helps investigate the persistently induced genes of *M. tb*, under consistent hypoxic conditions for a long time. The aerated exponential *M. tb* culture upon incubation for 4 h, 8 h, 12 h, 96 h, and 168 h under hypoxic conditions is followed by transcriptional profiles at each time point. Remarkably, 230 genes were initially not expressing until late and consistent hypoxic conditions of hypoxia, as after, 96 h and 168 h [20]. Hence, they can be correlated with the induction of late-stage hypoxia. In a previous study, *dosR* regulon was found to be induced within 2 h of hypoxia to control its initial stage in *M. tb* [21,22]. Data mining revealed that nearly 47 and 5 genes, induced in this model were common with nutrient starvation and Wayne hypoxic models respectively [16,19]. Precisely, *Rv0251c, Rv1805c, Rv1152, Rv2517c*, and *Rv3290c* were conserved in the obtained set of induced genes, among all the three models discussed previously.

### Drug persister model

To resist the wrath of antibiotics and the host immune system, bacteria evolves different strategies, for instance, an antibiotic-tolerant, persistent subpopulation develops within the bacterial population, but, it is afflicting to have a limited insight about these persister cells. In the drug persister model, an exponentially growing culture of *M. tb* is incubated with an antibiotic, D-cycloserine, capable of lysing the bacterial cells and used for isolating persister cells. RNA samples from culture aliquots, taken at various times, say, 7 or 14 days for transcriptomic studies. Furthermore, upon transcriptomic profiling, 1408 genes were found to be downregulated and 282 genes upregulated [23]. Thus numerous genes were downregulated and a majority of those were involved in growth and energy metabolism [23]. In-depth analysis suggested that five genes namely *Rv0251c, Rv1805c, Rv1152, Rv2517c*, and *Rv3290c* were common among the data obtained from the drug persister model, enduring hypoxic response, nutrient starvation, and Wayne hypoxic models. Later, Torrey et al. (2016) unraveled certain *M. tb* mutants that had a high persister formation tendency in both *in vitro* cultures and clinical isolates [24]. Upon comparison of these mutants by whole-genome sequencing and transcriptomic analysis from the *in vitro* cultures and clinical isolates, some genes involved in carbon metabolism, lipid biosynthesis, transcriptional regulators, and toxin-antitoxin system, implying that multiple pathways involve in *M. tb* persister formation and these high persister forming mutants may furnish in the relapse of tuberculosis infection [24].

### The granuloma model

Granulomas are the organized structures formed by infected macrophages and activated lymphocytes. These are the niche of the nonreplicating persistent bacilli where they survive and hide from stressful conditions like hypoxic, oxidative stress, nutrient starvation, and acidic pH. Thus, through *ex vivo* granuloma model, the host-mycobacterium interactions during dormancy and resuscitation could be understood. Loss of acid fastness, accumulation of lipids, and resistance to anti-TB drugs are the characteristic of *M. tb* harbored in human granulomas, which can be observed in this model also [25]. In this model, human peripheral blood mononuclear cells (PBMCs) growing in *M. tb* infected collagen matrix are extracted and incubated for 8 days, during which the lymphocytes aggregate around the infected macrophages to form the microgranuloma structure. Detailed investigations found that the granuloma model better emulates the human TB granuloma by compromising the ability of *tgs1* mutants to enter dormancy and *lipY* mutant to get out of dormancy [25].

### LivE model

*M. tb* adapts and survives within the lysosomes of activated macrophages, but limited information is available regarding the molecular players involved in this survival strategy. The LivE model, also called the Lysosomal *in vitro* exposure (LivE) model, assists in apprehending this survival strategy. The model includes exposure of *M. tb* to the soluble fraction of lysosomes extracted from activated murine macrophages [26]. Transcriptomic studies revealed that *M. tb* incubated with 20 µg/ml of lysosomal soluble fraction for 48 h led to upregulation of 264 and downregulation of 106 genes [27]. Moreover, comparison studies of the transcriptional profiles of LivE model with other stress models led to four genes (*Rv2036, Rv1472, Rv0251c*, and *Rv1956*) that were common among the nutrient starvation model, gradual hypoxic model, and the enduring hypoxic response model [27].

### Lipid-rich dormancy model

Lipids are crucial for pathogenesis of *M. tb* during its interaction with the host [28,29]. During infection, *M. tb* modulates lipid metabolism of itself as well as the host. *M. tb* induces a low-density lipoprotein response to promote the formation of foamy macrophages to ultimately facilitate the formation of caseous granuloma comprising triglycerides, lactosylceramides, and cholesterol [30–32]. Several *in vitro* stress models exist but none of them recapitulates the lipid-rich environment that presents within the granuloma. Lipid-rich dormancy model is the recent model developed to mimic the lipid environment present inside the tuberculosis granuloma. In this model, *M. tb* was separately *in vitro* cultured in presence of either lipid [33] or dextrose, followed by transcriptomic analysis at exponential phase, stationary phase, and nonreplicating persistence (NRP) phase, under both conditions [29]. The stationary and NRP phases are assumed to be the closest fit with the metabolic state of persistence. Upon differential gene expression analysis in the lipid environment, 368 genes were found, of which 185 genes were upregulated and 183 genes were downregulated [29]. Furthermore, six of them, namely, *Rv0678, Rv217c, Rv2393, Rv3159c, Rv3160c*, and *Rv3161c* were consistently expressed among all three phases and thus were designated as “the main core lipid response” set of genes [29].

## Functional aspects of genes established in *M. tb* persistence

**Figure 2.**
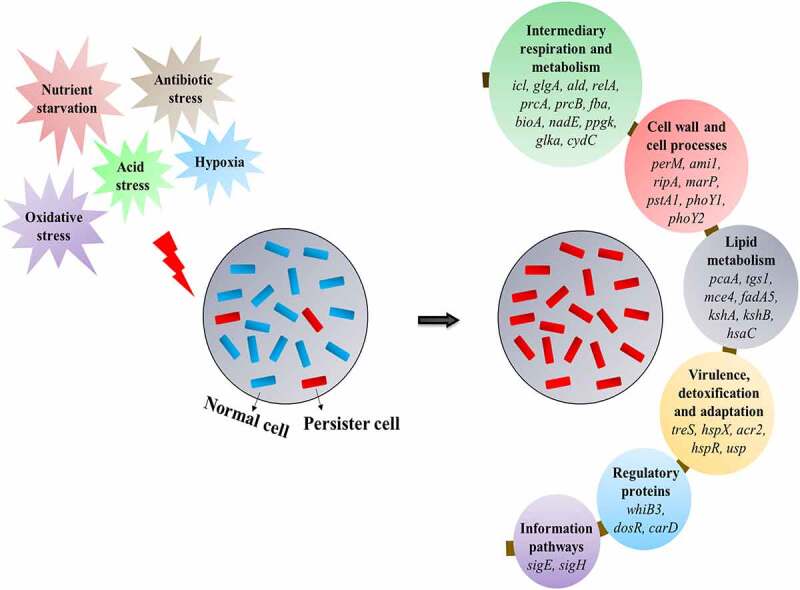
Stress conditions induce persistence in the *M. tb* that is brought by the interplay of different genes belonging to various essential pathways of the pathogen

As the *M. tb* cells acquire a persistent state or withstand unfavorable growth conditions, the integrated expression of several different molecular determinants from essential cellular processes, such as virulence, detoxification and adaptation, cell wall and cell process, intermediary respiration, lipid metabolism, and various regulatory pathways are needed. Here we unravel the genes that are experimentally proven (Figure 2) to be involved in the regulation of *M. tb* persistence, as shown in Table 2 and Table 3.

**Table 2.** Various genes of *M. tb* that are induced under mentioned stressful conditions and are known to be involved in persistence of the pathogen are listed

Rv no.	Gene name	Functional product	Function^2^	Induction conditions^3^	References
**Intermediary metabolism and respiration**					
Rv0467	*icl1*	Isocitrate lyase	Involved in glyoxylate cycle	Low pH, low oxygen, macrophage infections, *in vivo* TB granuloma conditions	[40,132,149,232]
Rv1212c	*glgA*	Putative glycosyl transferase	Probably involved in cellular metabolism	Low oxygen	[44,70]
Rv2780	*ald*	L-alanine dehydrogenase	Involved in cell wall synthesis	Low oxygen, nutrient starvation	[16,55,70]
Rv2583c	*relA*	Probable GTP pyrophosphokinase	Involved in the metabolism of ppGpp	Nutrient starvation, low oxygen	[56,60,61]
Rv2109c	*prcA*	Proteasome α-subunit	Protein degradation	Reactive nitrogen intermediates, oxidative stress	[66]
Rv2110c	*prcB*	Proteasome β-subunit	Protein degradation	Reactive nitrogen intermediates, oxidative stress	[66]
Rv0363c	*fba*	Fructose-1,6-bisphosphate aldolase	Involved in glycolysis	Low oxygen, stationary phase, change in carbon source	[69–71]
Rv1568	*bioA*	Adenosylmethionine-8-amino-7-oxononanoate aminotransferase	Bioconversion of pimelate into dethiobiotin	Stationary phase	[78,233]
Rv2438c	*nadE*	Glutamine dependent NAD synthetase	Biosynthesis of NAD	-	[80]
Rv2702	*ppgk*	Polyphosphate glucokinase	Phosphorylation of glucose by using polyphosphate or ATP	-	[87]
Rv0650	*glka*	Glucokinase	Predicted role in sugar metabolism and regulation	-	[87]
Rv1620c	*cydC*	ATP binding ABC transporter CydC protein	Involved in cytochrome biogenesis	Low oxygen, nitric oxide	[88,89]
**Virulence, detoxification, and adaptation**					
Rv0126	*treS*	Trehalose synthase	Biosynthesis of trehalose	-	[45]
Rv2031c	*hspX*	Heat shock protein	Proposed role in the maintenance of long-term viability or replication during latent or initial infections, respectively	Low oxygen, nutrient starvation, macrophage infection, stationary phase	[16,22,90–92,149]
Rv0251c	*acr2*	Heat Shock protein	Involved in the initial step of translation at high temperature	High temperature, nutrient starvation	[16,97,98,100]
Rv0353	*hspR*	Probable heat shock protein transcriptional repressor	Involved in the transcriptional repression of heat shock protein	High temperature	[100]
Rv2623	*usp*	Universal stress protein family protein TB31.7	Function unknown	Low oxygen, nitric oxide, macrophage infection	[21,103–107]
**Regulatory proteins**					
Rv3416	*whiB3*	Transcriptional regulatory protein WhiB-like WhiB3	Involved in transcriptional mechanisms	Low pH, nutrient, starvation, phosphate starvation	[108,109,171]
Rv3133c	*dosR*	Two component transcriptional regulatory protein	Regulatory part of two component system devR-devS	Low oxygen, nutrient starvation, nitric oxide	[16,22,103,112,117–119]
Rv3583c	*carD*	Transcriptional regulatory protein	Regulation of rRNA transcription	DNA damage and nutrient starvation	[57]
**Information pathways**					
Rv1221	*sigE*	Alternative RNA polymerase sigma factor	Promotes the attachment of RNA polymerase to transcriptional initiation site	Nutrient starvation, macrophage infections, high temperature, low pH, detergent stress	[16,99,100,122,124–126,132]
Rv3223c	*sigH*	Alternative RNA polymerase sigma factor	Regulation of thioredoxin cycling in oxidative stress response	Macrophage infections, high temperature, low oxygen	[100,132,234]
**Lipid metabolism**					
Rv0470c	*pcaA*	Cyclopropane synthase	Involved in the synthesis and modifications of mycolic acid	Low temperature	[140,235]
Rv3130c	*tgs1*	Triacylglycerol synthase 1	Involved in the synthesis of triacylglycerol	Low oxygen, low pH, low nutrient, high CO_2_	[143,144]
Rv3546	*fadA5*	Acetoacetyl-CoA thiolase	Involved in β-oxidations of side chains of cholesterol	Cholesterol, human macrophages	[147,149]
Rv3526	*kshA*	Oxygenase component of 3-ketosteroid 9α-hydroxylase	Involved in cholesterol catabolism	Nutrient starvation	[16,152]
Rv3571	*kshB*	Reductase component of 3-ketosteroid 9α-hydroxylase	Involved in cholesterol catabolism	Microaerophilic conditions, nitrosative stress	[152,236]
Rv3568c	*hsaC*	Extradiol dioxygenase	Involved in cholesterol catabolism	-	[153]
**Cell wall and cell processes**					
Rv0955	*perM*	Probable conserved integral membrane protein	Probably involved in cell division	Magnesium limitation, low pH	[155,160]
Rv3671c	*marP*	Membrane associated serine protease	Hydrolysis of peptides/ proteins at serine residue	Low pH, oxidative stress	[160,237]
Rv1477	*ripA*	Peptidoglycan hydrolase	Involved in hydrolysis of peptidoglycan	Low pH	[164]
Rv3717	*ami1*	Amidase	Involved in hydrolysis of peptidoglycan peptide stems	-	[164]
Rv0930	*pstA1*	Phosphate transport system permease protein	Involved in active transport of inorganic phosphate and substrate across the membrane	Nutrient starvation	[170]
Rv3301c	*phoY1*	Phosphate transport system transcriptional regulatory protein	Transcriptional regulation of inorganic phosphate	Nutrient starvation	[169,170]
Rv0821c	*phoY2*	Phosphate transport system transcriptional regulatory protein	Transcriptional regulation of inorganic phosphate	Nutrient starvation	[169,170]

**Table 3.** Functional analysis of *M. tb* persistence genes through mutational, deletion, and overexpression studies

Gene	Information from mutational and expression studies	References
**Intermediary respiration and metabolism**		
Rv0467 (*icl1*)^4^	Mutant displays attenuated persistence in activated macrophages and during chronic phase of mice infection	[40]
Rv1212c (*glgA*)	Inactivation reduces glucan content and mutant is unable to persist in chronic mice infection	[44]
Rv2780 (*ald*)^5^	Mutant shows delayed recovery from the non-replicating persistence state	[55]
Rv2583c (*relA*)	Deletion reduces long-term survival *in vitro* and persistence in chronic mice infection	[56,60,61]
Rv2109c (*prcA*)^6^	Needed for persistence in chronic infection of mice	[66]
Rv2110c (*prcB*)	Required for persistence in chronic infection of mice	[66]
Rv0363c (*fba*)^7^	Mutant shows attenuated persistence in chronically infected mice	[71]
Rv1568 (*bioA*)	Necessary for establishment of persistence in mice	[78]
Rv2438c (*nadE*)^8^	Inactivation reduces the long term survival *in vitro* and non-replicating persistence is observed	[80]
Rv2702 (*ppgk*)^9^ Rv0650 (*glka*)^10^	Double mutant has impaired persistence as demonstrated in chronically infected mice	[87]
Rv1620c (*cydC*)	Gene mutation enhances the killing of *M. tb* in isoniazid treated chronically infected mice	[88,89]
**Virulence, detoxification and adaptation**		
Rv0126 (*treS*)	Deletion mutant shows increased rate of mice survival	[45]
Rv2031c (*hspX*)	Deletion mutant shows increased bacterial growth upon tuberculosis infection in mice as well as in resting and activated macrophages *in vitro*	[90–92]
Rv0251c (*acr2*)^11^	Increased expression rapidly after entering the host cell during hypoxia and macrophage infection	[97,98]
Rv0353 (*hspR*)	Inactivation reduces the persistence ability	[100]
Rv2623 (*usp*)^12^	Deletion increases the bacterial growth and fails to establish a chronic tuberculosis infections in animals	[107]
**Regulatory proteins**		
Rv3416 (*whiB3*)	Mutant shows attenuated persistence in macrophages and guinea pigs model of infection	[109]
Rv3133c (*dosR*)^13^	Inactivation reduces the *M. tb* persistence in mice, guinea pigs, white rabbits and rhesus macaques	[117–119]
Rv3583c (*carD*)	Deletion reduces the bacterial survival in acute and chronic infection of mice	[57]
**Information pathways**		
Rv1221 (*sigE*)	Deletion mutant had reduced persistence in macrophages and in chronically infected mice	[99,124–126]
Rv3223c (*sigH*)	Deletion mutant causes increased apoptosis in non-human primate model	[234]
**Lipid metabolism**		
Rv0470c (*pcaA*)	Inactivation reduces the persistence in mice	[140]
Rv3130c (*tgs1*)^14^	Mutant decreases accumulation of TAG^15^ as well as antibiotic tolerance	[143,144]
Rv3546 (*fadA5*)	Attenuated phenotype in chronic phase of *M. tb* infection due to disruption of cholesterol catabolism	[147]
Rv3526 (*kshA*) Rv3571 (*kshB*)	Mutants are unable to persist in acute and chronic phase of mice infection	[152]
*mce4*	Required to persist in IFN-γ activated macrophages and in lungs of chronically infected mice	[82]
Rv3568c (*hsaC*)	Mutants unable to persist in lungs of guinea pig	[153]
**Cell wall and cell processes**		
Rv0955 (*perM*)	Required for persistence in chronically infected mice	[155]
Rv3571c (*marP*)^16^	Deletion lowers the persistence ability in the chronic phase of mice infection	[160]
Rv1477 (*ripA*)^17^	Mutant shows attenuated persistence in chronic phase of mice infection	[164]
Rv3717 (*ami1*)	Required for persistence in chronic phase of mice infection	[164]
Rv0930 (*pstA1*)^18^	Inactivation decreases the persistence in mice	[170]
Rv3301c (*phoY1*)	Disruption decreases the persistence in chronically infected mice	[169,170]
Rv0821c (*phoY2*)

### Intermediary respiration and metabolism genes

#### icl

Fatty acids and lipids are a significant source of carbon and energy for *M. tb* while infection as well as in persistence state [34,35]. This is one of the adaptation strategies for the long-term survival of persistent bacteria that enables the metabolic shift of carbon sources to C2 compounds produced by the β-oxidation of fatty acids [36]. Under these circumstances, the glyoxylate cycle increases significantly as an anaplerotic reaction that allows the C2 substrate to maintain the tricarboxylic acid (TCA) cycle [37]. Isocitrate lyase (Icl) is an enzyme of the glyoxylate cycle that catalyzes isocitrate to succinate and glyoxylate conversion. Expression of the *M. tb icl* gene was increased under oxygen-limiting conditions and during uptake in infected macrophages, suggesting that it may be involved in adaptation to unfavorable conditions favoring persistence in bacteria [38,39]. Deletion of *icl* gene in *M. tb* had a minor effect on the growth of bacteria in the acute infection phase however in the chronic infection phase the survival and growth of bacteria were severely impaired. The study also revealed that upon infection, the *icl* mutant of *M. tb* underwent strong attenuation, in activated macrophages, rather than in the resting macrophages [40]. Conclusively, a link could be established between the host immune status and the expression of *icl* gene in mycobacteria that suggests Icl is needed for the survival of *M. tb* during the persistent phase of infection. It is necessary for the bacterial survival in activated macrophages rather than that in resting macrophages.

#### treS *and* glgA

The outermost part of the *M. tb* cell wall is capsular, whose 80% extracellular polysaccharide is alpha glucan bound by alpha (1–4 linkage) with branching at every 5 to 6 residues by alpha (1–6 linkage) [41–43]. *glgA* gene encodes alpha-1,4-glycosyltransferase enzyme and the corresponding mutant *glgA* possess low glucan content in the capsule. This mutant was thus unable to persist within the infected mice, indicating that the complete capsule is needed for persistence [44]. Nevertheless, still, no direct evidence connecting the GlgA protein with the persistence of *M. tb*, but still under investigation. Another gene *treS* involved in the synthesis of trehalose from maltose [45]. Trehalose is a significant structural component of the cell wall glycolipid, as it forms trehalose-6, 6ʹ-dimycolate (TDM), which is ester-linked trehalose with two mycolic acid residues [46,47]. TDM has several functions implicated in the *M. tb* pathogenesis such as inhibition of phagosome to lysosome fusion that protects bacteria from acid-dependent macrophage killing [48] and conversion of NAD to NADH that results in the depletion of NAD-dependent enzyme activity during infection [49]. *treS* mutant of *M. tb* upon infection causes enhanced survival of mice relative to the wild type infected, suggesting the role of trehalose remodeling in the persistence of *M. tb* [45]. Probably, the need of TreS protein for persistence could be due to the increased need of trehalose or perhaps to catalyze the stored depots of trehalose to maltose followed by conversion to readily usable glucose [45]. Recent studies have shown that non-replicating *M. tb* uses trehalose as an adaptive strategy during hypoxic conditions by remodeling of trehalose metabolism and decreasing the synthesis of glycolipids such as trehalose monomycolate (TMM)/TDM [50–52]. Further Lee et al. (2019) states that drug-induced persistence includes remodeling of trehalose metabolism to increase the carbon flux toward the synthesis of glycolytic intermediates as well as pentose phosphate pathway intermediates that are the source of alternative biosynthetic energy molecules like ATP and NADPH along with antioxidants all for the survival of the bacilli during persistence [53].

#### ald

*ald* is one of the genes that encode alanine dehydrogenase and is induced under hypoxic conditions [54]. Ald is a multispecific enzyme that has two enzymatic activities, one is pyruvate reductive aminase activity, which catalyzes pyruvate to alanine and vice versa. The other activity is glyoxylate reductive aminase, which catalyzes the conversion of glycine to glyoxylate. Both enzymatic activities are coupled to oxidation of NADH thus forming NAD [54]. During hypoxic conditions, *M. tb* switches its energy sources from carbohydrates to fatty acids, and the glyoxylate cycle works as an anaplerotic reaction to synthesize four-carbon compounds as the substrate for β-oxidation of fatty acids [34]. There is no change in the survival of *ald* mutant under hypoxic conditions but there is a significant delay in the recovery of persistent bacteria upon reaeration, which is due to the altered NADH/NAD ratio [55]. It is also necessary to maintain the redox balance of persistent bacteria by regulating the NADH/NAD ratio. During reactivation, this NADH/ NAD ratio acts as a signal for the conversion of the non-replicating persistent state of bacteria to active growth and an optimal NADH/NAD ratio may be required for this transition, and if this ratio is not achieved, it may remain in a non-replicating persistent state.

#### relA

The stringent response is an adaptive strategy of mycobacteria to cope with numerous stress conditions such as nutrient starvation [56–58], oxidative stress [57], and stationary phase [58]. This stringent response is characterized by the accumulation of hyperphosphorylated guanine nucleotides like (p)ppGpp controlled by RelA. RelA is a bifunctional protein consisting of two enzymatic activities (p)ppGpp synthetase catalyzes the transfer of pyrophosphate from ATP to GDP and GTP led to the synthesis of ppGpp and pppGpp, respectively [56]. RelA has (p)ppGpp hydrolase activity that results in dissociation of pyrophosphate group from it to give GTP or GDP as by-products [59]. Inactivation of *relA* gene in *M. tb* causes failure of the pathogen for prolonged survival under *in vitro* culture conditions [56] and also hampers its ability to persist in the chronic infection phase of mouse model, suggestive of the importance held by RelA for persistence in the chronic phase of *M. tb* infection [60,61]. Previous studies reported microarray analysis of *relA* mutants under nutrient sufficient or deficient conditionsthat resulted in the increased expression of several transcripts, indicating their involvement of multiple cellular processes in the persistence of tuberculosis infection such as heat shock proteins, PE/PGRS family members, cell wall synthesis enzymes, transcriptional factors, and virulence factors [60], but the one specifically involved in persistence is not yet known. Insights into the role of RelA protein concerning mycobacterial persistence have to be gained and the individual enzymatic activity of this enzyme needs to be studied in-depth. The (p)ppGpp synthetase activity of RelA enzyme was abolished by point mutations in *M. tb*, which led to impaired growth and biofilm formation *in vitro* and abrogates *M. tb* to persist in the chronic phase of mouse infection [62]. However, the hydrolase activity of RelA enzyme is required both in the acute and chronic phases of infection indicative of the role of RelA in maintaining optimal levels of (p)ppGpp, which itself controls numerous cellular processes like GTP and ATP levels, DNA replication, translation machinery, and metabolism [62].

#### prcA *and* prcB

The *M. tb prcA* and *prcB* genes encode for the α-subunits and β-subunits of 20S proteasome respectively. It is anticipated that PrcBA is needed for the optimal *in vitro* growth of *M. tb* [63]. *M. tb* mutants lacking the proteasome accessory factors genes, such as *map* or *pafA* genes have low virulence, reflecting proteasome significance in the pathogenesis of *M. tb* [64,65]. The core of proteasome here plays crucial roles in defense against reactive nitrogen intermediates stress and also in the persistence of *M. tb* for chronic infections, as confirmed through genetic silencing of *prcBA* [66]. Thus *prcBA* is dispensable for the growth of *M. tb*. Although, when *prcBA* mutant was complemented by the active and mutated proteolytic proteasome, it states that proteasomal proteolytic activity is neither required for nitric oxide defense and nor for *in vivo* and *in vitro* growth of *M. tb* [67]. Conversely, proteasomal proteolytic activity is indispensable for the long-term survival of *M. tb in vitro* and chronic phase of mice infection. Further studies indicate that nitric oxide is not culpable for the attenuation of *M. tb prcBA* mutant in the chronic phase of infection, but some other mechanisms that control the mycobacterial persistence facilitated by proteasomal proteolysis. Intriguingly, *M. tb*, which lacks *prcBA* was unable to survive *in vitro* under nutrient starvation and stationary phase conditions and was failed to persist *in vivo* under the same conditions [67]. Previous studies in *E. coli* indicates that under nutrient starvation conditions there is an adaptation strategy for the survival of bacteria by increasing the degradation of ribosomal proteins through Lon protease, which has led to the availability of amino acids for the synthesis of new enzymes that regulate the essential cellular processes [68]. Likewise, *M. tb* proteasome core genes may be crucial to maintaining long-term persistence in the host by regulating the turnover of proteins and amino acid supply.

#### fba

It encodes the class II fructose-1,6-bisphosphate aldolase (FBA), which reversibly catalyses the cleavage of fructose-1,6-bisphosphate to produce glyceraldehyde-3-phosphate and dihydroxyacetone phosphate in the glycolysis cycle. *M. tb*, both replicating and persistent, expresses this enzyme *in vitro* under stressful conditions like low oxygen tension, stationary phase, and change of carbon sources and is needed for glycolysis and gluconeogenesis [69]. It was found to be induced under *in vivo* tuberculosis infections in mice and guinea pigs. Furthermore, persistent *M. tb* bacilli have increased *fba* gene expression under low oxygen tension conditions as an adaptation strategy [70]. Depletion of *fba* gene in *M. tb* led to strong attenuations in the ability to reside in the mouse lungs and spleen while acute and chronic infections implying that this protein plays role in growth and persistence [71]. However, it is still unclear how this protein influences persistence mechanisms.

#### bioA

Biotin synthesis in *M. tb* starts with pimeloyl-CoA, a reaction that occurs through the actions of BioF, BioA, BioD, and BioB in four enzymatic steps [72]. It serves as an indispensable component required for the oxidation of carbon dioxide in acyl-CoA carboxylases and pyruvate carboxylase, both being a part of fatty acid metabolism and gluconeogenesis respectively [73,74]. Using S-adenosyl methionine (SAM) as an amino group donor, BioA catalyzes the second step of biotin synthesis by transamination of 7-keto-8-aminopelargonic acid (KAPA) to 7,8-diaminopelargonic acid (DAPA) [75–77]. In the absence of exogenous biotin, the *bioA* mutant fails to produce biotinylated proteins in *M. tb* that are essential for the fatty acid biogenesis, resulting in the elimination of mutant in biotin-free media. Conditional silencing of this mutant after the establishment of infection indicates that de novo synthesis of biotin is needed to sustain infection and persistence in a mouse model of TB infection [78]. As a result, the inactivation of BioA enzyme can be used as a target to clear off *M. tb* during both the acute and chronic phase of infection.

#### nadE

Nicotinamide adenine dinucleotide (NAD) biosynthesis is important since it regulates several processes including NAD cofactor pool, redox balance, respiration, central carbon, and energy metabolism. NAD biosynthesis is through the conversion of nicotinamide mononucleotide precursors to nicotinamide dinucleotide intermediates through two enzymes NaMN adenylyl transferase (NadD) and NAD synthetase (NadE) [79]. Inactivation of *nadE* gene led to mutant *M. tb* with a substantial reduction in the ability for *in vitro* growth and non-replicative persistence, induced under nutrient limitations or low oxygen tensions and is subsequently eliminated in the acute and chronic phases of infection in mice [80]. Further, Rodionova et al. (2014) had used the protein knockdown approach to target NadD and NadE enzyme that resulted in the diminished of the NAD cofactor pool, which then prevents the metabolic flux in NAD(P)-dependent pathways including, central carbon metabolism and energy production [81]. Taken together, these studies indicate that NadE is a potent persistence target whose inactivation leads to the loss of both the replicative as well as non-replicative persister bacilli.

#### Ppgk *and* glka

The metabolic adaptations of *M. tb* are crucial for establishing and maintaining chronic infections in the host, and existing pieces of evidence suggest that fatty acids and lipids are the primary energy sources during infection [82–86]. Nevertheless, the significance of other carbon sources during infection and persistence is not known till date. PPGK (Polyphosphate glucokinase) and GLKA (Glucokinase) are the two functional glucokinases that metabolize glucose by conversion of glucose to glucose-6-phosphate. Deletion of *ppgk* gene led to a mutant with mild attenuations while growing in the presence of glucose and this indicates that the mutant possesses the ability to use glucose as an energy source during infection [87]. However, in absence of both glucokinases, PPGK and GLKA, the *M. tb* mutant is impaired to persist in the mice chronic infection model, thus implying that the *M. tb* relies on the phosphorylation of glucose to access glucose as an energy source to survive under the chronic infection [87].

#### cydC

The *cydC* encodes an ATP-binding protein ABC transporter that is necessary for the assembly of cytochrome bd oxidase and is upregulated in presence of hypoxia and nitric oxide *in vitro* and during the chronic phase of mice infection [88]. Deletion of *cydC* had attenuated the *M. tb* growth and survival during its transition to the chronic phase of mice infection as well as increases the clearance of *M. tb* in chronically infected mice treated with isoniazid, relative to wild type [88,89]. Further, this might be possible that isoniazid activation by catalase releases nitric oxide and inhibits cytochrome bd oxidase activity, enhancing isoniazid-mediated *M. tb* killing [89]. But how *cydC* gene affects the mycobacterial persistence and the enhanced killing of *M. tb cydC* mutant by isoniazid in chronically infected mice is not yet clear.

### Virulence, detoxification, and adaptation

#### hspX *and* acr2

HspX and Acr2 proteins belong to the alpha crystalline-like protein family. Under hypoxic conditions, the transcript levels of *hspX* or *acr* (Rv2031) gene increases in *M. tb*, which indicates that this protein is required for adaptation to the hypoxic conditions within the host [90]. Deletion of *hspX* gene increased the growth rate of mutant in comparison to the wild type *M. tb*, in the macrophages and in mice infection. This suggests that it is required for the active growth and transitioning from the log to stationary phase in bacteria [90–92]. Under the stationary phase of bacteria, this protein was assumed to stabilize the *M. tb* cell wall by its chaperonin activity, which influences bacterial growth rate directly or indirectly [93]. This protein is also immunodominant in humans indicative of its involvement in the mechanisms by which *M. tb* evades the immune response to establish infection, as evidenced by increased expression of it in the stationary phase [94]. Later, overexpression of HspX protein in Bacille Calmette–Guérin (BCG)-immunized mice (BCG:HSP) and the alone BCG-immunized mice, both kinds were protected to equal extent upon tuberculosis infection. But, in immunodeficient mice infected with BCG:HSP persist longer compared to control BCG strain, implying that HspX could be a potential vaccine candidate [95]. Further, HspX was used as a part of subunit vaccine adjuvanted with N, N’-dimethyl-N, N’-dioctadecyl ammonium bromide (DDA) and trehalose-6,6ʹ-dimycolate (TDM) elicited a stronger humoral and T-cell mediated immune response, and could boost the BCG-primed immune response against *M. tb* infection in mice [96]. Another protein Acr2 (Rv0251c), a member of the alpha crystalline protein family, was induced in murine macrophages and *in vitro* under several stress conditions, including heat shock, high dose of nitric oxide, detergent stress, peroxide stress, and palmitic acid [97]. There is a strong induction of *acr2* gene expression shortly after the phagocytosis of *M. tb* by quiescent murine and human macrophages [97,98]. In addition, deletion of *acr2* gene had unaffected the *in vitro* growth of *M. tb* but persisted in IFN-γ activated human macrophages [98]. The *acr2* gene expression is regulated through two proteins: SigE – controlled by heat shock and oxidative stress [99], and HspR – heat shock regulator [100]. Therefore, the early expression of this gene suggests that Acr2 appears to be an immunodominant antigen (Ag) that elicits a strong early immune response to *M. tb* infection [98].

#### hspR

*hspR* is a gene that exists in an operon containing *hsp70, grpE*, and *dnaJ* genes. These heat shock proteins are found to have functional interactions with Hsp70 [101]. It acts as a repressor that controls the expression of *hsp70* operon and *clpB* gene of *M. tb* [100,102]. Deletion of *hspR* gene in *M. tb* led to the reduction in the survival of this mutant in bone marrow-derived macrophages and chronically infected mice model that suggests metabolic adaptation in the *hspR* mutant that could be beneficial for mycobacteria survival in acidified phagosome during the chronic phase of infection [100]. Overexpression of Hsp70 protein combined with inactivation of *hspR* mutant leads to an increased Ag expression per bacteria that might generate a stronger immune response and adopt this mutant as an attractive target to strengthen the host immune responses during persistent infection of *M. tb* [100].

#### usp

Rv2623 is a universal stress protein of *M. tb* and a member of the dormancy regulon, which has increased expression under low oxygen tension and high nitric oxide [21,103,104]. It is also highly induced in mouse and human macrophages along with its increased expression in the lungs of chronically infected mice [105,106]. Furthermore, deletion of Rv2623 gene in *M. tb* shows hypervirulent phenotype, as evidenced by increased mortality, histopathology, and bacterial development. Besides, *in vitro* overexpression of Rv2623 in *M. tb* results in bacterial growth retardation as compared to the parental strain. Together, this information suggests that Rv2623 is essential for the establishment of persistent infection by regulating the *M. tb* growth under *in vitro* and *in vivo* conditions [107]. More importantly, point mutations in the ATP binding site of Rv2623 exhibit normal bacterial growth as parental strain, implying that Rv2623 regulates the mycobacteria growth in an ATP-dependent manner [107]. Nevertheless, the accurate mechanism by which it is involved in persistence is still unknown.

### Regulatory proteins

#### whiB3

WhiB3 is a redox-sensitive transcriptional regulator with four iron-sulfur (Fe-S) cluster that responds to host generated oxygen and nitric oxide to maintain the redox homeostasis [108]. In addition, it encourages the growth of persistent bacilli under different stress conditions such as low pH and nutrient starvation [108,109]. In previous studies, it was reported that deletion of *whiB3 M. tb* and *Mycobacterium bovis* (*M. bovis)* had little effect on the growth of two animal models, mice and guinea pigs, though there was a reduction in colony-forming counts (CFU) of *M. bovis whiB3* mutants in guinea pigs model of infection [110]. In contrast, a recent study has shown that *whiB3* mutant of *M. tb* causes *in vivo* attenuation in the lungs of guinea pigs and has an impaired ability to survive in macrophage [109]. However, the mechanisms by which *M. tb* senses the different stresses and modulates the host immune system to promote bacterial persistence are not well understood. Transcriptomic analysis reveals that the functioning of *whiB3* gene facilitates *M. tb* adaptation in infected macrophage by controlling the expression of virulence, lipid production, redox homeostasis, cell wall remodeling and metabolic adaptation in response to available carbon sources whereas host microarray indicates WhiB3 protein of *M. tb* regulates the expression of host cell cycle genes and DNA damage checkpoints [111]. Conclusively, WhiB3 protein appears to be a redox sensor that controls polyketide expression by modulating bioenergetics metabolism in response to the host environment. During *M. tb* infection, activated macrophage releases polyketide and cyclomodulin, which arrests the host cell cycle and modulate the immune response, allowing long-term survival of persistent bacilli [111].

#### dosR *and* dosS

DosR (Dormancy survival regulator) comprises a regulon of more than 50 genes, that are activated in response to gradual depletion of oxygen and under nitric oxide stress inside granuloma, allowing the transition of active replicating *M. tb* bacteria to dormant state to cope up with these stresses and increase its long-term survival in the host [21,103,112,113]. Additionally, this regulon is crucial for the resuscitation of dormant *M. tb* bacilli to the active replicating state upon normoxic growth conditions [114]. The DosR regulon includes genes essential for persistence such as *tgs1* gene (triglyceride synthase), *hspX* (alpha crystalline family heat shock protein gene), and *Rv2623* (universal stress protein). The DosR regulon had been extensively studied, in *M. tb*, it is phosphorylated by two molecules DosS and DosT, under different growth-restricting conditions such as hypoxia, nitric oxide, ascorbic acid, and carbon monoxide [112,115]. The majority of research is diverted on *dosR*, as its expression is enhanced upon infection in macrophages, in various animal models, and during the latent stage of infection [112]. Inactivation of *dosR* in *M. tb* has previously shown no effect upon the bacterial burden and histopathology in different mice strains including, C57BL/6, DBA2, C3He/FeJ, and C3HeB/FeJ [20,116]. Whereas, other studies in C57BL/6 mice, guinea pigs, white rabbits, and rhesus macaques documented a strong attenuation in the growth of *dosR M. tb* mutant and impaired histopathology [117–119]. Overall, these findings demonstrate that DosR-regulated Ags delay the adaptive immune response during infection by inhibiting the T-cell response, emphasizing the significance of DosR regulon in modulating the host immune response to facilitate the *M. tb* persistence [119]. However, the mechanism by which DosR adapts the *M. tb* in the hypoxic condition is unclear. Yang et al. (2018) substantiated that in hypoxic conditions, DosR is deacetylated, thus resulting in increased DNA binding ability, which eventually affects its regulon, allowing *M. tb* to rapidly adapt to hypoxic conditions and persist for longer periods [120]. In addition to DosR, DosS is also important for the persistence of *M. tb*. The *dosS* mutant of *M. tb* in macrophages is severely attenuated compared to the wild type *M. tb* and other *dos* mutants [121]. This is due to the induction of TNF-α and IFN-γ leading absence of phagosomal maturation arrest. The dosR mutant of *M. tb* is not attenuated within macrophages, indicating DosS can perform functions independent of DosR [121]. Supporting the above findings, the dosS mutant of *M. tb* was severely attenuated in C3HeB/FeJ mice and rhesus macaques but could grow under microaerophilic and hypoxic conditions, suggesting that the attenuation was not due to hypoxia [121]. Recent evidence suggests that 36 out of 51 dos genes are upregulated in presence of cholesterol as a carbon source both in actively replicating *M. tb* as well as in hypoxic conditions [113]. Furthermore, the induction of *tgs-1* gene is inhibited relative to other *dos* genes in response to *prpR* deletion using cholesterol in the growth medium, suggesting that PrpR rather than DosR regulates TAG synthesis utilizing cholesterol as a carbon source [113].

#### carD

CarD is a transcriptional regulator protein that regulates the transcription of rRNA genes in mycobacteria by binding to the β-subunit of RNA polymerase [57]. It is induced in presence of stress conditions such as oxidative stress, DNA damage, and nutrient deprivation. Further, deletion of the *carD* gene in *M. tb* causes bacterial survival to be attenuated in both the acute and chronic phases of mouse infection, indicating the CarD is required not only in bacterial replication but also requisite for *M. tb* persistence [57].

### Information pathways

#### sigE

Extra cytoplasmic RNA polymerase sigma factor (SigE), one of the best-studied sigma factors in *M. tb*, is encoded by the *sigE* gene. It acts as a central regulator of *M. tb* stress response that induces under a variety of stressful environments including pH stress, heat shock response, oxidative stress, detergent stress, vancomycin mediated cell surface stress, and during growth in human macrophages [122]. Deletion of *sigE* gene in *M. tb* triggered its persistence in the lungs of *M. tb* aerosol-infected mice due to delay in death time of this mutant [123]. Since the *sigE* mutant was unable to block phagosome maturation in macrophages, inactivation of *sigE* gene led to the decreased viability of this mutant in both naïve and activated macrophages [99,124]. This mutant was strongly attenuated, indicating that it was unable to grow in mice and generate a heightened immune response than wild type *M. tb* [125,126]. Under low phosphate concentrations and chemical stress during infection, the *sigE* mutant is needed for the synthesis of *M. tb* capsular polysaccharides [127]. Additionally, the microarray analysis of *sigE* mutant, indicating decreased transcript levels of classical heat shock proteins, transcriptional regulators, and enzymes involved in fatty acid oxidation [99]. Previous studies reported that it functions as a bistable switch that may be involved in persister formation during hypoxic growth arrest [128,129]. However, it is unclear how important *sigE* deletion is for persistence during antibiotic treatment. To answer this problem, Pisu et al. (2017) reported that *sigE* mutant killed much faster than wildtype *M. tb* in presence of various antibiotics including vancomycin, gentamicin, rifampin, streptomycin, isoniazid, and ethambutol, which revealed that fewer persisters remaining in the sigE mutant culture [130].

#### sigH

SigH is an extracytoplasmic sigma factor of *M. tb* that is induced under different stress conditions such as oxidative stress, cell wall damage, phagocytosis, enduring hypoxia, heat shock response, and reaeration [131–133]. Possibly, it has a role in the reactivation of non-replicating persistent *M. tb* to actively growing *M. tb* [134]. Deletion of *sigH* gene in *M. tb* fails to induce granulomatous pathology despite *M. tb* replication in mice [135]. However, *sigH* gene mutant of *M. tb* in the non-human primate (NHP) model induces highly organized human-like granulomatous lesions and generates a heightened immune response to the bacilli upon infecting the host macrophages as compared to the wildtype *M. tb* [136]. This heightened immune response was manifested by the increased level of β-chemokine secretion and chemotaxis of inactivated monocytes and increasing the extent of apoptosis. Thus, SigH appears to be crucial for modulating the host immune response during *M. tb* infection by secreting molecules that interact with the host immune machinery and modulate chemotaxis and apoptosis. Ultimately, it would significantly promote the long-term survival of *M. tb* that facilitates the persistence and spreading of initial infection because chemotaxis is required for the migration of activated immune cells to the site of infection and apoptosis is an innate mechanism that is required for the clearance of *M. tb* [137,138]. To strengthen this point, a study by, Du et al. (2016) reported that inactivation of SigE and SigH transcription factors in *M. tb* leads to impaired ability to recover from persistence [139].

### Lipid metabolism

#### pcaA

*pcaA* gene encodes cyclopropane synthase that has methyltransferase activity, one of the enzymes involved in the modification of mycolic acid present in the cell wall of mycobacteria. This enzyme is essential in the cord formation and synthesis of the proximal cyclopropane ring of alpha mycolic acid in *M. tb* and BCG [140]. Deletion of *pcaA* gene led to the impaired cyclopropanation, altered colony morphology, and inability to form serpentine cords that result in enhanced replication in the initial phase of infection as compared to wild type *M. tb* and inability to persist in the chronically infected mice. Ultimately, this suggests the significance of PcaA protein in the development of persistent chronic infection [140]. Now, the question comes in mind as to how *pcaA* gene expression influences *M. tb* persistence. Sequentially, autophosphorylation of serine/threonine-protein kinase (STPK) by some host signal in phagosomes led to the phosphorylation of PcaA at two residues, threonine-168 and threonine-183 that results to the several outcomes, including it inhibits the formation of cyclopropane rings in the cell wall of *M. tb*, as shown by the lack of di-cyclopropanated alpha mycolic acid, restricts intramacrophage replication, and prevents phagosome-lysosome fusion [141]. Consequently, it was discovered that PcaA is needed for the survival of mycobacteria during persistent chronic infection because it regulates the fusion of late endosomes to lysosomes.

#### tgs1

The family of triacylglycerol synthase contains 15 genes, one of which, *tgs1* gene, is responsible for the accumulation of triacylglycerol (TAG) in *M. tb* under various stress conditions [142,143]. As *M. tb* is exposed to several stress conditions such as hypoxia, nitric oxide, acidic pH, and low nutrient, TAG accumulates as an energy reserve in the dormant state of *M. tb*, allowing it to persist for long periods [142,144]. Interesting enough, a recent study has proved that *M. tb* exploits the host TAG by releasing fatty acid that accumulates in the form of TAG in *M. tb* during infection [145]. In contrast to wild type, a mutant of *tgs1* gene in *M. tb* abolishes TAG accumulation and unable to tolerate antibiotics under stress conditions [143,144].

#### mce4

Cholesterol is also a lipid-based carbon and energy source in addition to fatty acids utilized by *M. tb* for growth under nutrient deprived conditions within macrophages during infection, as reported by several studies [146]. Though genome of *M. tb* lacks the genes for cholesterol synthesis, however the genes needed for scavenging the host cholesterol such as for its transport and catabolism are present [82]. Mce4 (Mammalian cell entry protein) is one of the well-studied cholesterol import system of *M. tb*, required for the acquisition of host cholesterol. This transporter system is reported to be dispensable for growth, both in resting macrophages and during establishment of mice infection. However, needed for *M. tb* growth in IFN-γ-activated macrophages, wherein it persists for longer periods in lungs of chronically infected animals [82].

#### fadA5

*fadA5* (Rv3546) encodes β-ketoacyl-CoA thiolase that carries the β-oxidations of side chains of cholesterol. *fadA5* synthesis is regulated by cholesterol and KstR protein such that the former upregulates and the latter represses its expression [147,148]. *fadA5* was found to be upregulated in the *in vitro* cultures of *M. tb* supplemented with cholesterol along with human macrophages [149] and mice lungs [150] infected with *M. tb*. This observation indicates the involvement of cholesterol during *in vivo M. tb* growth. FadA5 enzyme of *M. tb* catalyzes the conversion of cholesterol to androst-4-ene-3,17-dione (AD) and 1,4-androstadiene-3, 17-dione (ADD) by two successive β-oxidations [147]. These intermediate metabolites can be utilized by the *M. tb* as source of carbon and energy during *in vitro* and chronic phase of mouse lung infection that facilitates *M. tb* persistence. *fadA5 M. tb* mutant has attenuated phenotype due to the disruption of the cholesterol metabolism that is requisite for the persistent phase of *M. tb* infection [147,148].

#### kshA and kshB

*kshA* and *kshB* genes of the *M. tb* cholesterol catabolic pathway encode for 3-ketosteroid 9α-hydroxylase (KSH) enzyme, which is required for opening of sterol ring of cholesterol [151]. It catalyzes the conversion of ADD to 9-hydroxy-1,4-androstadiene-3, 17-dione steroid intermediate for the utilization of cholesterol by *M. tb* during infection. Deletion of these two genes in *M. tb* impairs the ability of bacteria to persist for longer periods in the stationary growth phase under the microaerophilic conditions [152]. Thus, these are indispensable for the growth as well as persistence in both the resting and activated macrophages. *kshA* and *kshB* mutants are unable to grow and persist in acute as well as chronic phase of murine infection [152]. These attenuated mutants cannot metabolize cholesterol and 4-androstadiene-3, 17-dione steroid intermediates as carbon and energy source during infection [152].

#### hsaC

*hsaC* gene encodes an iron-dependent extradiol dioxygenase (HsaC) that catalyzes the final step of cholesterol degradation in *M. tb*. HsaC catalyzes the extradiol ring cleavage of DHSA (3,4-dihydroxy-9,10-seco-nandrost-1,3,5(10)-triene-9,17-dione) to produce 4,9-DSHA (4,5–9,10-diseco-3-hydroxy-5,9,17-trioxoandrosta-1(10),2-diene-4-oic acid) [153]. Studies carried with immunocompromised mice infected with either *hsaC* deletion mutant of *M. tb* or the parental strain reveals prolonged survival of the former. Likewise, in guinea pigs *hsaC* mutant of *M. tb* shows less granulomatic lesions, spreads relatively slow in the spleen and is unable to persist within lungs [153]. This attenuated phenotype is owed to the disruption of cholesterol catabolism and the undesirable toxicity of catechols or quinones. Thus, *M. tb* uses cholesterol, whose catabolism begins at the initial stage of infection before the onset of the host adaptive immune response, which then becomes key nutrient at the chronic or later phase of *M. tb* infection [153,154].

### Cell wall and cell processes

#### perM

PerM (rv0955) is an integral transmembrane protein, consists of ten transmembrane helix, and is indispensable for the persistence of *M. tb*. Initially, it has been shown that disruption of *perM* gene through transposon insertion directed to severe growth defects in the chronic phase of mouse infection but mild attenuation in the acute phase [155]. Therefore, the question arises what exactly is the role of PerM protein and how it regulates the mechanism of persistence in *M. tb*. Further research on this mutant, indicates that PerM is a component of the mycobacterial divisome complex that allows mycobacteria to divide by stabilizing FtsB. Remarkably, it maintains the level of FtsB, a conserved protein divisome that is essential for septum formation [156–158]. It is required in host mimicking stress conditions to maintain the level of FtsB that is crucial to control the cell division at the chronic mice infection, but dispensable in the acute phase of infection [159].

#### marP, ripA, *and* ami1

The periplasmic serine protease MarP (Mycobacterium acid resistance protease) is present in *M. tb*. Under acidic conditions, *marP* mutant of *M. tb* was unable to survive because it failed to maintain intracellular neutral pH and gets severely attenuated to an extent that it shows impaired growth during the initial (acute) phase of infection and thus is unable to persist in the chronic phase of mouse infection [160]. Upon acid stress, the *marP* mutant bacteria forms elongated cells along with multiseptal chains indicative of their role in cell separation at low pH [161]. The mechanism by which MarP protein is required for the *M. tb* survival in acidic conditions found in the host phagosomes has been described. First, MarP senses acid stress and triggers inactive RipA cleavage, resulting in active RipA with peptidoglycan hydrolase activity [161]. Consequently, RipA regulates *M. tb* peptidoglycan hydrolysis a process required for cell wall homeostasis and bacterial cell separation and also important for *M. tb* survival in acidic environments. RipA and RipB (Resuscitation promoting factor interacting partners) are peptidoglycan endopeptidase that cleave the peptide bond between D-glutamic acid and diaminopimelate of peptidoglycan peptide stem [162]. Deletion of *ripA* in *Mycobacterium smegmatis* forms normal cells as they are in wild type *M. smegmatis* under regular growth conditions, but under acidic conditions, it results in elongated and multiseptal cells [161,163]. Nonetheless, in the case of *M. tb*, RipA is indispensable for cell division and cell growth during *in vitro* normal growth conditions as well as during persistent chronic phase of infection, but deletion of *ripB* gene did not affect cell division, though this enzyme is required in the absence of RipA [164]. It is needed not only for cell separation during cell division by peptidoglycan degradation at the septum but also enables incorporation of new peptidoglycan content during cell elongation by cleaving the peptidoglycan at the polar region [164]. This endopeptidase is found in the septa and poles region of *M. bovis* BCG [165], where it interacts with other cell wall enzymes including RpfB, a lytic transglycosylase [166] and PonA1, a peptidoglycan synthase [167]. In *ripA* mutant, it also affects the enzymatic activity of its interacting partner that might be helpful to control the peptidoglycan remodeling during the chronic phase of infection. RipA protein is necessary for the persistence of *M. tb*, but the precise mechanisms are unascertained. Intriguingly, a study by Shariq et al. (2021) demonstrates that RipA modulates the metabolic reprogramming, as well as, inhibits autophagy and apoptosis of macrophages in conjunction with TLR-4 surface immune receptors [168]. Based on these facts, a significant survival strategy of *M. tb* involves employing RipA for replicating within macrophages, subduing the host immune defense. Another peptidoglycan-modifying enzyme, Ami1, which belongs to the L-alanine amidase family cleaves the peptide stem from the glycan’s backbone at N-acetylmuramic residue. While Ami1 is needed for mycobacterium persistence during chronic mouse infection, it is not required for cell division under *in vitro* growth conditions. However, it is necessary for normal cell growth in *M. smegmatis* [164]. The most probable reason for Ami1 importance in chronic phase infection is that it aids the cell division of a subpopulation that can withstand in host-driven stress conditions.

#### pstA1, phoY1, *and* phoY2

PhoY1 and PhoY2 are the two important proteins that play crucial roles in the formation of *M. tb* persisters. Previous studies stated that phoY2 but not phoY1, is needed for *in vitro* culture of *M. tb* and mouse model of tuberculosis infection [169], but a subsequent study found that both PhoY1 and PhoY2 are requisite for *M. tb* growth and survival in chronically infected mice [170]. However, the implication of PhoY proteins in *M. tb* persistence is unclear. To solve this puzzle, a recent study found that PhoY proteins function as a mediator between PstA1 phosphate transporter and SenX3-RegX3 two-component system to control the phosphate sensing signal transduction mechanism that somehow involved in persister formation. Under inorganic phosphate limiting and *in vitro* growth conditions, deletion of the *phoY1* and *phoY2* genes, as well as the *pstA1* gene, mediate the activation of RegX3, indicating a decrease in persister formation [170]. In addition, *phoY* and *pstA1* mutants in *M. tb* were more susceptible to rifampicin rather than isoniazid in aerosol-infected mice model of tuberculosis [170]. Furthermore, disrupting the *regX3* gene increases the persister frequency under phosphate-limiting conditions [171–173]. As a result of these efforts, it appears that both PhoY and PstA1 proteins are required to inhibit the persister formation in *M. tb* by activating RegX3 under phosphate-limiting conditions or by disrupting the signaling between PstA1 and SenX3-RegX3.

## Impact of therapeutics on Mycobacterium persistence

Current advancements in understanding molecular determinants involved in *M. tb* virulence and pathogenesis, as well as how bacteria evades the host immune defense and persist for long periods, can provide detailed information about the dynamic relationship of human host and pathogen. With this information, researchers may be able to develop new therapeutics that target different stages of tuberculosis pathogenesis, such as the active and latent stages of infection, to completely eradicate the bacteria and improve defense against transmission. Table 4 lists various therapeutic regimens for active and latent tuberculosis detection.

**Table 4.** Numerous potential drug and vaccine candidates for *M. tb* that have been developed recently with their respective stages of clinical trials are listed

Therapeutic regimens^19^^,^^20^	Formulation of therapeutic regimens	Clinical trials
**Latent TB drug**		
DOLPHIN IMPAACT4TB	Isoniazid and rifapentine	Phase I/II
IMPAACT P2001	Isoniazid and rifapentine	Phase I/II
TBTC Study 35	Isoniazid and rifapentine	Phase I/II
A5279/BRIEF TB	Isoniazid and rifapentine	Phase III
A5300B/I2003/PHOENIx	Delamanid	Phase III
CORTIS	Isoniazid and rifapentine	Phase II/III
TB-CHAMP	Levofloxacin	Phase III
TBTC study 37/ASTERoid	Rifapentine	Phase II/III
V-QUIN trial	Levofloxacin	Phase III
WHIP3TB	Isoniazid and rifapentine	Phase III
P1078 IMPAACT/ TB APPRISE	Isoniazid	Phase IV
**Vaccine**		
Ad5Ag85A^21^	Replication-deficient human adenovirus serotype-5 vector expressing Ag85A antigen of *Mycobacterium tuberculosis*	Phase I
H1:IC31^22^	Recombinant fusion protein ESAT-6 and Ag85B of *M. tb* with IC31 adjuvant (TLR-9 agonist)	Phase I
H4:IC31^22^	Recombinant fusion protein TB10.4 and Ag85B of *M. tb* with IC31 adjuvant (TLR-9 agonist)	Phase I
AEC/BC02^22^	Ag85B antigen and Fusion protein of CFP-10 and ESAT-6 of *M. tb* with CpG adjuvant	Phase I
AERAS-422^22^	Recombinant BCG vaccine expresses Ag85A, Ag85B, and Rv3407 antigens mixed with perfringolysin	Phase I
AERAS-402^21^	Adenovirus serotype 35 (Ad35) expressing Ag85A, Ag85B, and TB10.4 antigens of *M. tb*	Phase I
ChAdOx185A – MVA85A^21^	Replication-deficient chimpanzee adenovirus/ modified vaccinia Ankara virus vector expressing Ag85A antigen of *M. tb*	Phase I
GamTBvac^22^	Fusion of two *M. tb* proteins (Ag85A and ESAT6-CFP) with dextran-binding domain in DEAE dextran and CpG (TLR-9 agonist) adjuvant	Phase IIa
ID93:GLA-SE^22^	Recombinant fusion protein (ID93) comprises four antigens involved in virulence (Rv2608, Rv3619, Rv3620) and latency (Rv1813) of *M. tb* with GLA-SE adjuvant (emulsion of glucopyranosyl lipid and MPL)	Phase IIa
MTBVAC^23^	Attenuation via deletions of *phoP* and *fadD26* genes in live strain of *M. tb*	Phase IIa
RUTI®^24^	Liposome coated cell wall fragments of *M. tb* bacteria	Phase IIa
TB/FLU-04L^21^	Attenuated influenza viral vector expressing Ag85A and ESAT-6 antigens of *M. tb*	Phase IIa
Gates MRI-TB01-201^23^	Live attenuated *Mycobacterium bovis*	Phase IIb
DAR-901 booster^24^	Heat killed whole cell or extract of *Mycobacterium obuense*	Phase IIb
M72/AS01_E_^22^	Recombinant *M. tb* fusion protein of two antigens Rv1196 and Rv0125 mixed with AS01_E_ adjuvant (a liposomal fraction of saponin-fraction QS21and TLR-4 ligand MPL-A)	Phase IIb
H56:IC31^22^	Recombinant *M. tb* fusion protein of three antigens expressed during initial (Ag85B), late (ESAT-6) and throughout the *M. tb* infection (Rv2660c) with IC31 adjuvant (a TLR-9 agonist)	Phase IIb
BCG Revaccination^23^	Live *M. bovis* BCG vaccine	Phase IIb
Vaccae^TM24^	Whole cell or extract of *Mycobacterium vaccae*	Phase III
VPM1002^23^	Live recombinant *M. bovis* with urease C deletion and lysteriolysin insertion	Phase III
MIP/Immuvac^24^	Heat killed *Mycobacterium indicus pranii*	Phase III

### Diagnostics

It is a strenuous task to diagnose the non-replicating bacilli as there is no suitable diagnostic test to do so; hence it is not possible to assess the level of persistence in tuberculosis-infected asymptomatic individuals. Since latent tuberculosis infection (LTBI) is linked with low tissue bacterial burden, any diagnostic scheme based on identifying the bacteria or its biological components is more challenging. The cellular immune response resulting from mycobacterial Ags, LTBIs are usually diagnosed rather indirectly. LTBI is diagnosed most often by blood-based IGRA (Interferon gamma release assay) and skin-based TST (tuberculin skin test). TST evaluates delayed-type hypersensitivity against purified protein derivatives (PPD) of mycobacterial cells *in vivo*, with the outcome being the extent of the skin induration region over 2–3 days [174]. Tuberculin PPD is made of the protein precipitate of mycobacterial culture filtrates, that comprises of conserved chaperone proteins (constitutes nearly 60%) among mycobacterial species like heat shock proteins (HspX), 10-kDa chaperonin GroES, and 60-kDa chaperonin 1 (GroEL) [175]. However, the presence of conserved proteins in PPD impedes the TST to distinguish among various *M. tb* infections, for instance, environmental non-tuberculous mycobacteria, and BCG vaccination. In contrast, IGRA being a blood-based test identifies the IFN-γ release from the sensitized T-cells exposed to mycobacterial ESAT-6 and CFP-10 *in vitro* [174]. The IGRA test assesses the cell-mediated immune response, wherein the outcome depends upon the IFN-γ levels generated from the circulating effector memory cells [176] and the effector T cell frequency. As a result, neither BCG vaccination nor exposure to non-tuberculous mycobacteria affects IGRA results. However, the individuals with high incidence of *M. tb* exposure like healthcare professionals are shown to give variable IGRA result values. This implies that either there is poor test repeatability or reinfection-inducing reversion as a consequence of ongoing exposure to mycobacterial Ags [177]. Furthermore, in low-risk populations, erroneous conversions seem to be more frequent with IGRAs than with TST [178]. Improving on existing TST and IGRA to generate better tests is one way for addressing this issue. The C-Tb hypersensitive skin test sensitive to recombinant ESAT-6 and CFP-10 proteins is an example of this approach [179]. The high specificity of IGRA and the minimal cost of TST are meant to be combined in this diagnostic test. Additionally, two more commercially available diagnostic tests, namely QuantiFERON (QFT) and QFT TB Gold in tubes (QFT-GIT), are composed of long peptide derivatives of ESAT-6 and CFP-10 Ags. Another diagnostic test called as QFT-TB Gold Plus (QFT-Plus), is developed that contains both long peptides derived from ESAT-6 and CFP-10 Ags to elicit specific CD4^+^ T-cell response and short peptides for IFN-γ release by CD4^+^ and CD8^+^ T-cells [180]. It is observed that addition of peptides to stimulate CD8^+^ T-cells can assist to distinguish LTBI from active TB [181,182]. In people with LTBI, the QFT-Plus assay has a greater correlation with increased *M. tb* exposure than the QFT-GIT assay [180]. T-SPOT.TB is another diagnostic test that is based on ESAT-6 and CFP-10 Ags of *M. tb*. The ELISPOT technique used in this assay quantifies the level of IFN-γ produced by the T-cells, which necessitates an expensive reader machine, appropriate software, and specialized trained staff, thus limiting its applicability in developing countries [183]. According to studies, the metabolism of *M. tb* keeps changing during infection, which is reflected by the variable expression of immunodominant Ags. This allows to distinguish among different stages of infection and to assess the risk of active TB progression, for diagnostic purposes [184]. Numerous studies revealed that mycobacterial Ags Rv2628, Rv1737, Rv2029c, and Rv2004 elicit CD4^+^ and CD8^+^ T-cells and hence IFN-γ in LTBI individuals compared to those with active tuberculosis [174]. Likewise, a recent study categorized certain proteins under active and latent tuberculosis specific biomarkers. These include Alr, BfrA, EspR, TrpG, and TreY in active tuberculosis and HspR, NarG, PonA1 and PonA2 in latent tuberculosis [185–187]. Furthermore, assessment of these proteins would assist either in discovery of newer therapeutics or diagnostic markers to facilitate delineation between the active and latent stage of tuberculosis.

### Vaccine

*M. tb* uses numerous tactics to hamper the host immune defenses. The persistence phenomenon empowers the bacteria to stay alive within the host in a latent state, in which infected individuals are asymptomatic and are unable to transmit the disease. Nevertheless, this latent or persistent stage bacterium is primarily responsible for the recalcitrance of chronic tuberculosis infection as when the bacteria encounter favorable conditions it resumes to form the wild type population of bacteria. It was verified in human studies that there is a gap in the expression of antigenic repertoire in individuals infected with active and latent tuberculosis [160,161]. Furthermore, most of the proteins involved in bacterial persistence were revealed through different omics approaches under various stress conditions imposed by the host have already been addressed in this review, could be used to design a vaccine against the latent stage of tuberculosis [7]. To avert reactivation on latent tuberculosis individuals, these candidates should induce a T-cell response and neutralizing antibodies against the proteins involved in persistence. There are varied T-cell responses elicited to potential TB vaccines, as well as discrepancies in the relative strengths of immune responses, including the T_H_1, T_H_17, and CD8^+^ responses, both in humans and animal models [188]. Candidates for TB vaccines produce both CD8^+^ as well as CD4^+^ T-cell response having distinct characteristics, even though these responses have variations between studies in animals and clinical trials. BCG challenge induces multifunctional T_H_1 and T_H_17 responses in mice and nonhuman primates and partially protects against *M. tb* [189–191]. The intensity or multifunctional profiles of BCG-specific T-cells could not provide protection against pulmonary TB in the infants of South Africa [192]. Intriguingly, BCG-specific IFN-γ response is associated with a lower incidence of tuberculosis under the same circumstances [193]. Also, BCG-induced immunity and efficacy gradually fade, especially in high TB prevalence areas [194–196]. In comparison to BCG, the other live-attenuated mycobacterial vaccines like AERAS-422, VPM1002, and MTBVAC elicit stronger immune response, encompassing robust multifunctional CD8^+^ and CD4^+^ T-cell responses that enhance immunity in mice [197–200] but these responses are not substantially better from those elicited by BCG during clinical trials [201–204]. Although in infants the Ag-specific CD4^+^ T-cell responses evoked by MTBVAC were found to be stronger than those induced by BCG but, the CD8^+^ T-cell responses were low. Subunit vaccine candidates as BCG boosters stimulate Ag-specific CD4^+^ and CD8^+^ T-cell responses in mice leading to improved immunity. Nevertheless, noncoherent protection in NHPs (Ad5Ag85A, MVA85A, H1/H4/H56, and AERAS-402) [205–210] while modest immune responses in BCG-vaccinated infants (ID93, AERAS-402, M72, and MVA85A) [211–216] relative to adults were found for certain candidates. Based on clinical trials, MVA85A does not significantly enhance protection against *M. tb* infection regardless of heightened T_H_1 and T_H_17 responses [217,218], similarly, AERAS-402 triggers multifunctional CD8^+^ and CD4^+^ T-cells that are incapable of recognizing the *M. tb*-infected cells [219]. In contrast to BCG-induced responses, mycobacterial adjuvant subunit vaccine formulations preferentially stimulate co-expression of IL-2 cytokine and multifunctional CD4^+^ T-cell populations associated with increased protection in mice [220–222]. However, the relevance of these CD4^+^ T-cell populations in human protection remains undetermined. Also, the significance of variations in the immunogenicity of subunit vaccine antigenic components is yet to be determined [223,224]. As a therapeutic vaccine, MIP (*Mycobacterium indicus pranii*) shows promising results in animal models infected with *M. tb*, although in clinical studies, it shows obscure advantages in individuals infected with TB [225–227]. Though, there is a lack of correlates of protection for infants and adults from tuberculosis infection. The antigenic heterogeneity during an infection and the lack of host biomarkers are some of the factors that prevent tuberculosis vaccine trials from being successful. In conclusion, the development of a TB vaccine can be initiated through the use of relevant animal and human disease challenge models, synchronization between the outcomes of preclinical and clinical vaccine trials, as well as the assessment of vaccine candidates through advanced clinical trials.

### Drug

The current drug regimen of tuberculosis is effective in killing drug-susceptible tuberculosis, but it is often ineffective in eradicating drug-resistant and drug-persistent tuberculosis infections. Persistent *M. tb*, which is non-replicating in nature, adapts in the host stress microenvironment through reducing metabolism and increasing phenotypic antibiotic tolerance [228]. Furthermore, under favorable growth conditions, persistent *M. tb* has the potential to convert into the active population of *M. tb* so it is critical to target the persistent state to prevent active TB progression. Till now, there is not even a single drug available on the market that explicitly targets the persistent mycobacteria. There lies an imperative requirement of potential drugs that can target both actively replicating as well as non-replicating characteristics of mycobacteria to fully wipe out the drug-resistant and persistent *M. tb*. The new potential drug candidate should possess the following characteristics: high safety with few side effects, shorter therapy length, oral bioavailability, and it should also target the persistent, multidrug-resistant, as well as extensively drug-resistant tuberculosis. Recent research has discovered some effective inhibitors that target both active and dormant tuberculosis bacteria, as listed in Table 5, such as N-(pyridine-2-yl methyl)-2-(4-(quinolin-4-yl) piperazin-1-yl) acetamide, 1-((4-methoxyphenyl) sulfonyl)-4ʹ, 5ʹ-dihydrospiro [piperidine-4, 7ʹ-thieno [2, 3-C] pyran] and biphenyl amide (DG70). The first two compounds inhibit the Lysine-ε aminotransferase enzyme that is responsible for reversibly catalyzed the transamination of lysine into α-ketoglutaric acid, while the third compound, DG70, act as a respiration inhibitor and inhibits the dimethyl menaquinone methyltransferase (MenG) enzyme that inhibits the final step of menaquinone synthesis [229–231]. Synergistic activity of these inhibitors, as well as existing drugs with established mechanisms of action could shorten treatment time and make highly effective against the treatment of tuberculosis.

**Table 5.** Inhibitors that target genes involved in *M. tb* persistence are enlisted with their respective inhibitory (IC_50_) concentrations

Inhibitors	Target protein	Inhibition (IC_50_)^25^	References
1-((4 methoxyphenyl)sulfonyl)-4′,5′-dihydrospiro[piperidine-4,7′-thieno[2,3-*c*]pyran]	Lat	1.04 ± 0.32 µM	[229]
N-(pyridine-2-yl methyl)-2-(4-(quinolin-4-yl) piperazin-1-yl) acetamide		1.04 µM	[230]
Biphenyl amide DG70	MenG	> 80 µg/ml	[231]
2-Vinyl-d-isocitrate	Icl	10 ± 1.3 µM	[238]
3-Nitropropionate		14.7 ± 1.8 µM	[239]
3-Bromopyruvate		17.5 ± 1.0 µM	
Itaconic acid		29.4 ± 4.1 µM	
Methyl-4-(4-methoxyphenyl)-4-oxobut-2-enoate		250 ± 7.0 µM	
Lead 1	Ald	35.54 ± 0.0033 µM	[240]
Lead 2		80.37 ± 0.010 µM	
Lead 3		51.53 ± 0.0048 µM	
Lead 4		36.84 ± 0.030 µM	
Lead 5		73.84 ± 0.0232 µM	
Compound X9	RelA	4.8 µM	[241]
8-Hydroxyqunoline carboxylic acid	Fba	10 ± 1 µM	[242]
Compound 1		0.0016 µM	[243]
Compound 2		0.185 µM	
Compound 3		0.12 µM	
Compound 4		0.31 µM	
Compound 1ʹ8		0.013 µM	
Compound 2ʹ8		0.17 µM	
4-(Benzothioazole-2-yl) pentenoic acid	BioA	153 nM	[244]
Dihydro-4-pyridone		3.9 ± 1.2 mM	[245]
Compound A35		88.16 µM	[246]
Compound A36		28.94 µM	
Compound A65		114.42 µM	
Urea sulfonamide analog 4 f	NadE	90 ± 5 µM	[247]
Phenylcoumarin derivative	DosR	< 26.2 µM	[248]
Artemisinin (HC101A)		10 µM	[249]
HC102A		10 µM	
HC103A		10 µM	
HC102A	DosS	1.9 µM	
HC103A		0.5 µM	

However, many obstacles such as lack of suitable biomarker to measure its efficacy, inadequate information about the mechanism of persistence in mycobacteria, and lack of established animal models, slow down the drug-development process from the identification of lead compounds to the time until it is launched in the market as an approved drug. Conclusively, the main goal of this review article is to explore more about the persistence mechanism in mycobacteria through its crucial molecular players involved in various essential processes, including lipid metabolism, intermediary respiration and metabolism, virulence, detoxification, adaptation, and cell wall synthesis.

## Discussion and future remarks

The main reason for failures in treating clinical tuberculosis is the persistence of mycobacteria, which is transiently tolerant to drugs used in tuberculosis therapy. It provides a clear indication that conventional therapeutic options, such as the BCG vaccine and a combination of tuberculosis drugs, are insufficient to eradicate tuberculosis infection. In the past decade, the researches on bacterial persistence have made significant progress. However, persistence remains to be a major issue to public health, so detailed investigation about the biology of persisters and their mechanisms is required to achieve better clinical results. Overall, there is an urgent need for the development of therapeutic options aimed at both active and latent *M. tb* bacteria. Although the development of some therapeutics, such as bedaquiline drug and M72-based vaccine had shown promising results, but the lag at the level of comprehensive understanding about the tuberculosis pathogenesis impedes the development of better therapeutics and diagnostics.

Research studies have found that multiple genes and regulatory pathways of both host and mycobacteria are responsible for persistence and their eventual relapse to actively-replicating wild type populations. The cohesive efforts of persister enrichment through approaches like fluorescence-activated single cell sorting and laser capture microdissection and analysis techniques such as time lapse microscopy, microfluidics technique, omics technologies, and next-generation sequencing, can provide robust information to understand the mechanism of persister formation and their reactivation. Gene network analysis and system biology techniques could assist to unravel the ways through which the molecular determinants of bacterial cells interact in stressful environments giving rise to numerous persister phenotypes, who even differ at their frequency level under different stress conditions.

Intriguingly, the *in vitro* model system that mimics the different stress conditions imposed by the host immune system on the pathogen provides a screening approach that recognizes the significance of *M. tb* genes in the adaptive response during infection. Various *in vitro* and *ex-vivo* models have been developed to imitate the stress conditions faced by the *M. tb* inside the host during infection. These models are established to understand the mycobacterial factors responsible for adaptation of bacteria to persistent state. But notable limitation of these stress models is that their information is not significant compared to the *in vivo* scenario in clinical and animal models. For instance, nutrient availability studies in the context of persistence *in vitro* differ considerably to those of *in vivo* conditions. As a matter of fact, different micro niches such as adipose tissues, macrophages, and mesenchymal stem cells of the human body are exploited by the mycobacterial cells to hide away from the host immune response. These hidden bacterial cells then utilize host fatty acids and cholesterol as carbon and energy sources to persist for long-term within the host. Thus, unraveling the relationship between the bacterial persistence and host metabolism can present newer avenues to develop therapeutics and diagnostics. The molecular determinants that are identified to be implicated in mycobacterium persistence are well described in this review, which gives certain insights into the biology of persister formation and its reactivation in mycobacteria. In addition, this information will also facilitate the development of biomarkers that could demarcate between active and latent tuberculosis to promote the molecular diagnosis of tuberculosis.

Another uncharted field of research includes the study of persistence in context of host microbiota, which is currently deemed interesting by many researchers. Although, bacterial persistence is assumed to be adverse as it is the reason for the recalcitrance of chronic infections, but persistence could be favorable for the survival as well as diversity of healthy microbes of host microbiome under conditions like pathogenic or viral infections, change in diet, age, and antibiotic treatment. Although, it is not substantiated that persister cell formation occurs in the host microbiome however, it is important to comprehend the possibilities of utilization of the persistence phenomenon to reestablish the host microbiota.

For *M. tb* a major obstacle to comprehend the mechanism of persister formation and subsequent relapse is the unavailability of clinical specimens and tissue samples from individuals with latent or persistent tuberculosis. In addition, there is a lack of imaging techniques and diagnostic tools to classify the persister cells in clinical specimens. Thus, due to the lack of thorough information about the persistent tuberculosis infection, creating clinically relevant persistence models is an arduous task. So, it is imperative to build well-designed specimen banks to preserve the clinical samples from individuals with latent or persistent tuberculosis, both prior or upon reactivation. This data is pivotal to develop therapeutic interventions for successful identification and targeting of the persistent or latent tuberculosis bacteria before it relapses in the host.

## Data Availability

The authors confirm that the data support the findings of this study are available within the article.

## Notes

1. Prepared by mixture of Purecol collagen solution, Dulbecco’s phosphate buffer saline, fibronectin, and sodium hydroxide

2. Putative and known function

3. These genes are primarily presented based on induction conditions that have been associated with increase persistence in *Mycobacterium tuberculosis*.

4. Icl: Isocitrate lyase

5. Ald: Alanine dehydrogenase

6. Prc: Proteasome core

7. FBA: Fructose-1,6-bisphosphate aldolase

8. NAD: Nicotinamide adenine dinucleotide

9. PPGK: Polyphosphate glucokinase

10. GLKA: Glucokinase

11. Acr: Alpha crystalline

12. Usp: Universal stress protein

13. Dos: Dormancy survival

14. Tgs: Triacylglycerol synthase

15. TAG: Triacylglycerol

16. Mar: Mycobacterium acid resistance

17. Rip: Resuscitation-promoting factor interaction partner

18. Pst: Phosphate-specific transport

19. Sable SB, Posey JE, Scriba TJ. Tuberculosis vaccine development: progress in clinical evaluation. Clinical microbiology reviews. 30 October 2019;33(1):e00100-19.

20.
https://www.who.int/publications/i/item/9789240013131

21. Recombinant live-attenuated or replication-deficient virus vector candidates

22. Mycobacterial fusion protein in an adjuvant formulation

23. Live-attenuated mycobacterial vaccine candidates

24. Mycobacterial killed, whole-cell, or extract vaccine candidates

25. IC_50_: The concentration of chemical/drug/inhibitor required for 50% inhibition of a specific biological or biochemical function.
